# Inhibition of cell cycle-dependent hyphal and biofilm formation by a novel cytochalasin 19,20‑epoxycytochalasin Q in *Candida albicans*

**DOI:** 10.1038/s41598-023-36191-4

**Published:** 2023-06-15

**Authors:** Kwanrutai Watchaputi, L. A. Channa Bhathiya Jayasekara, Khanok Ratanakhanokchai, Nitnipa Soontorngun

**Affiliations:** 1 Excellent Research Laboratory for Yeast Innovation, Division of Biochemical Technology, School of Bioresources and Technology, King Mongkut’s University of Technology Thonburi (KMUTT), Bangkok, 10150 Thailand; 2 Excellent Center of Enzyme Technology and Microbial Utilization, Pilot Plant Development and Training Institute, King Mongkut’s University of Technology Thonburi (KMUTT), Bangkok, 10150 Thailand

**Keywords:** Transcriptional regulatory elements, Biofilms

## Abstract

Biofilm-mediated drug resistance is a key virulence factor of pathogenic microbes that cause a serious global health threat especially in immunocompromised individuals. Here, we investigated the antihyphal and antibiofilm activity of 19,20‑epoxycytochalasin Q (ECQ), a cytochalasin actin inhibitor isolated from medicinal mushroom *Xylaria* sp. BCC1067 against *Candida albicans*. Remarkably, 256 µg/ml of ECQ inhibited over 95% of *C. albicans* hyphal formation after 24 h-treatment. Combined ECQ and lipid-based biosurfactant effectively enhanced the antihyphal activity, lowering required ECQ concentrations. Hyphal fragmentation and reduction of biofilm biomass, shown by SEM and AFM visualization of ECQ-treated biofilms, were well corelated to the reduced metabolic activities of young and 24 h-preformed *C. albicans* biofilms. Induced intracellular accumulation of reactive oxygen species (ROS) also occurred in accompany with the leakage of shrunken cell membrane and defective cell wall at increasing ECQ concentrations. Transcriptomic analyses via RNA-sequencing revealed a massive change (> 1300 genes) in various biological pathways, following ECQ-treatment. Coordinated expression of genes, associated with cellular response to drugs, filamentous growth, cell adhesion, biofilm formation, cytoskeleton organization, cell division cycle, lipid and cell wall metabolisms was confirmed via qRT-PCR. Protein–protein association tool identified coupled expression between key regulators of cell division cyclin-dependent kinases (Cdc19/28) and a gamma-tubulin (Tub4). They coordinated ECQ-dependent hyphal specific gene targets of Ume6 and Tec1 during different phases of cell division. Thus, we first highlight the antihyphal and antibiofilm property of the novel antifungal agent ECQ against one of the most important life-threatening fungal pathogens by providing its key mechanistic detail in biofilm-related fungal infection.

## Introduction

Despite being part of human microbiome, *Candida* is one of the most widespread opportunistic pathogens and the most challenging to treat, especially in individuals with impaired immunity such as HIV, cancer, or organ transplant patients^1,2^. *C. albicans* is a polymorphic fungus, presenting many types of morphologies including the budding yeast form, pseudohyphae, hyphae, chlamydospores and opaque cells^3^. Hyphae and the opaque cells could evade the host immune system, and the hyphal form is necessary to invade host tissues^4,5^. *Candida* spp. can adhere to epithelial or endothelial surfaces as well as implanted medical devices by forming biofilms, which is an important virulence factor in promoting the progression and persistence of fungal infection. It is well established that *Candida* biofilms are resistant to many clinically-used antifungal agents, including the widely used drugs amphotericin B and fluconazole^6–9^. Due to drug resistance, those clinical drugs can also be of limited use. This not only provokes attention of the public heath, government and communities but also calls for urgent research studies on novel and effective antibiofilm agents.

Biofilms greatly contribute to persistent chronic infections as they shelter the pathogens, prevent the access of antifungal drugs, protect against host’s immune cells and other external stresses^10^. The first and most critical step of biofilm formation is the adhesion of yeast cells to surfaces and to each other to form initial biofilm layer which generally takes at least 1–2 h in the in vitro systems. After adhesion, cells proliferate and form an anchor layer that provides primary stability to the biofilm. Biofilm maturation typically takes 24 h in in vitro systems^11^. The fungal plasma membrane not only acts as a protective barrier against antifungals but also contributes to virulence through various dynamic processes such as secretion of virulence factors, cell wall construction, endocytosis, nutrient uptake and invasive hyphal morphogenesis^12^. The production of hyphae is related to biofilm growth as hyphal morphogenesis increases adhesion maintenance in the developing biofilm^13^. In addition, expression of genes encoding membrane-bound drug efflux pumps ATP-binding cassette (ABC) and major facilitator superfamily (MFS) transporters are reported to be upregulated during the development of biofilm into intermediate and mature stages, therefore supporting biofilm-mediated drug resistant mechanism^14^.

Natural products are still the most important source for the discovery of new and potential fungicidal agents, including biofilm inhibitors. Cytochalasans are a type of fungal metabolites produced through polyketide synthase/nonribosomal peptide synthase (PKS/NRPS) biosynthesis by the filamentous fungi in the family *Xylariaceae* (Ascomycota)^15^. Previous studies report that cytochalasans are natural product that found in abundance and possess a wide range of important biological activities^16^. Some examples include cytochalasin 6^17^, cytochalasin 10^18^, cytochalasin 11^18^, cytochalasin 12^19^, 19,20-epoxycytochalasin C^20^, phenochalasin C^20^ and chaetoglobosin A^21^ with the ability to inhibit biofilm formation of the pathogenic bacterium *Staphylococcus aureus*. Among others, the cytochalasin compound 19,20‑epoxycytochalasin Q (ECQ) has been reported to exhibit antifungal activity against the model yeast *Saccharomyces cerevisiae*^22,23^. Mechanisms of action of ECQ involved RAS and Srv2-associated actin aggregation, alteration of drug efflux transporters and loss of plasma membrane integrity^22^. Cell death is eventually caused by ECQ through the inhibition of actin depolymerization and excessive reactive oxygen species (ROS) generation^22^. The *erg6* homozygous deletion mutant with more permissive membrane is hypersensitive to ECQ where it interferes with lipid biosynthesis by lowering ergosterol and triacylglycerol levels^23^. The ECQ-treated Δ*erg6* mutant also has increased actin aggregation and lipid droplet assembly^23^. ECQ may also interfere with lipid homeostasis and other cellular processes such as endocytosis and the activity or localization of drug efflux pumps, thereby amplifying its antifungal activity.

This work aimed to further investigate the inhibitory mechanism of ECQ, isolated from the medicinal mushroom *Xylaria* sp. BCC 1067^24^, against *C. albicans* hyphal growth at different stages of biofilm formation. Using the high-throughput next generation sequencing technique RNA-seq, we also uncovered the underlying virulence factors whose expressions are suppressed by ECQ treatment and revealed key cell cycle proteins, involving in the yeast to hyphal transition, as the potential antifungal drug targets.

## Results and discussion

### ECQ inhibited *C. albicans* hyphal formation

**Figure 1:**
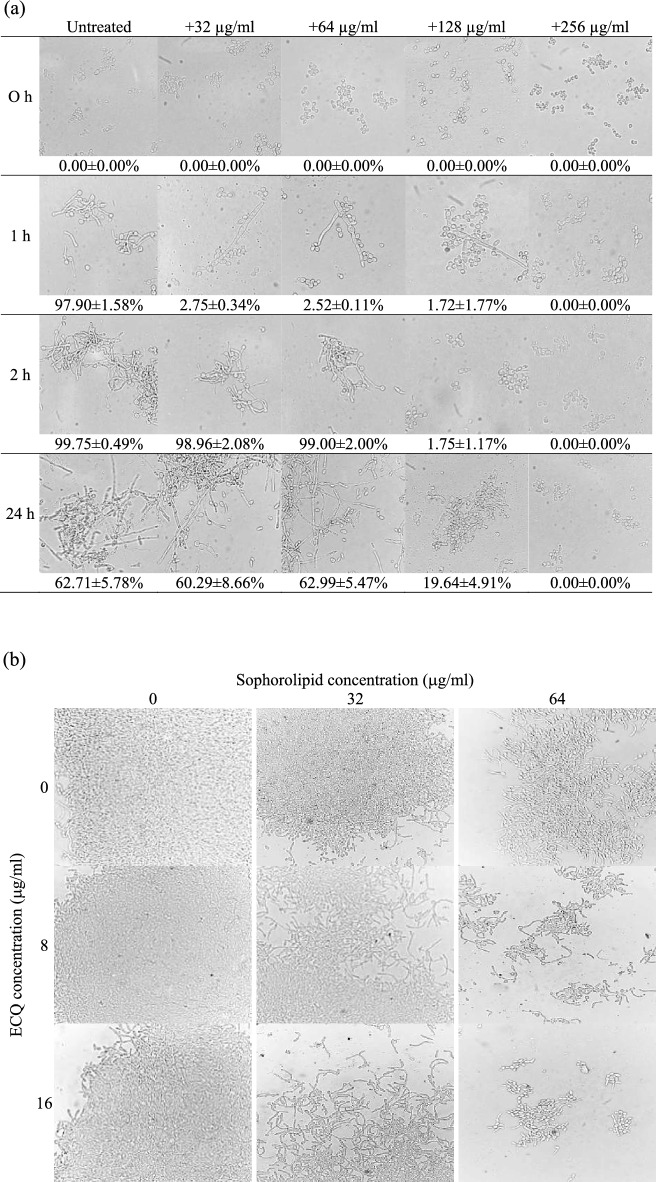
Effect of ECQ and ECQ in combination with sophorolipids against hyphal formation of *C. albicans*; (**a**) effect of ECQ in various concentrations against hyphal formation of *C. albicans* at 37 °C for 0, 1, 2 or 24 h and (**b**) effect of ECQ in combination with sophorolipids against hyphal formation at 5 h. Cells were observed using a microscope at 400 × magnification. Numbers represent the percentage of hyphal cells normalized with total cells (N > 120 cells).

*Candida albicans* has the ability to switch from yeast to hyphal growth, which is considered one of the most important steps in biofilm formation^25^. In this work, we first investigated the effect of ECQ treatment on hyphal formation of *C. albicans*. Hyphal formation was induced in RPMI-1640 medium containing a hyphal inducer (10% fetal bovine serum, FBS) and ECQ at 0, 32, 64, 128 and 256 μg/ml and incubated for 0 to 24 h. After 2 h of incubation at 37 °C, vigorous hyphal formation was observed in the untreated condition. A concentration-dependent inhibition of hyphal development was shown by ECQ, which began to inhibit hyphal production at 128 μg/ml and entirely inhibited at the higher concentration of 256 μg/ml (Fig. 1a). At 24 h, numbers of *C. albicans* in hyphal form were decreased by approximately 36% in the untreated condition with the presence of some budding cells. Treatments with 128 and 256 μg/ml of ECQ could inhibit hyphal cells by approximately 19% and 100%, respectively, at 24 h (Fig. 1a). Overall, ECQ displayed good ability to inhibit hyphal formation in *C. albicans*.

### Combination of ECQ and sophorolipids enhanced the anti-hyphal activity

ECQ exerted potent anti-hyphae activity, but its ability to generate ROS and cytotoxicity were causes for concern of antifungal treatment. ECQ was previously reported cytotoxicity against human epidermoid carcinoma (KB) and human breast cancer (BC-1) which exhibited IC_50_ of 0.72 and 20 μg/ml, respectively^26^. To reduce cytoxicity of ECQ, drug combination offers a promising strategy. Biosurfactants are considered potential candidates due to their low toxicity and being widely used as safe emulsifier in food industry^27^. The microbial derived-surfactant sophorolipids, obtained from *Starmerella riodocensis* GT-SL1R sp. nov. strain, is recently reported to show effective anti-hyphae and biofilm activity against *C. albicans* at concentrations of less than 1000 μg/ml^28^. Moreover, approximately 50% of *C. albicans* 90-min-old biofilm and mature biofilm were decreased in treatment with sophorolipids at concentration 125 μg/ml and 500 μg/ml, respectively^28^. We were thus interested to observe the anti-hyphal effect of combining ECQ and sophorolipids. After 5 h of 64 μg/ml sophorolipids treatment, hyphae appeared shorter, compared to untreated condition (Fig. 1b). Importantly, the combination of 8 or 16 μg/ml of ECQ and 64 μg/ml of sophorolipids exerted potent ability to inhibit hyphal formation of *C. albicans*, compared to untreated condition. Combined ECQ and sophorolipids effectively inhibited hyphal growth of *C. albicans* and lowered its cytotoxicity to sub-inhibitory concentration of < 20 µg/ml (Fig. 1b), therefore suggesting lipid-based formulation of ECQ. Despite promising antihyphal activity of ECQ-sophorolipids combination, the exact mechanisms for antimicrobial activity of ECQ or sophorolipids remain unclear. Thus, we must first explore their individual mechanism of action prior to the assessment of potential synergistic antifungal effect and formulation.

### ECQ reduced the *C. albicans* metabolic activity and biofilm formation

**Figure 2:**
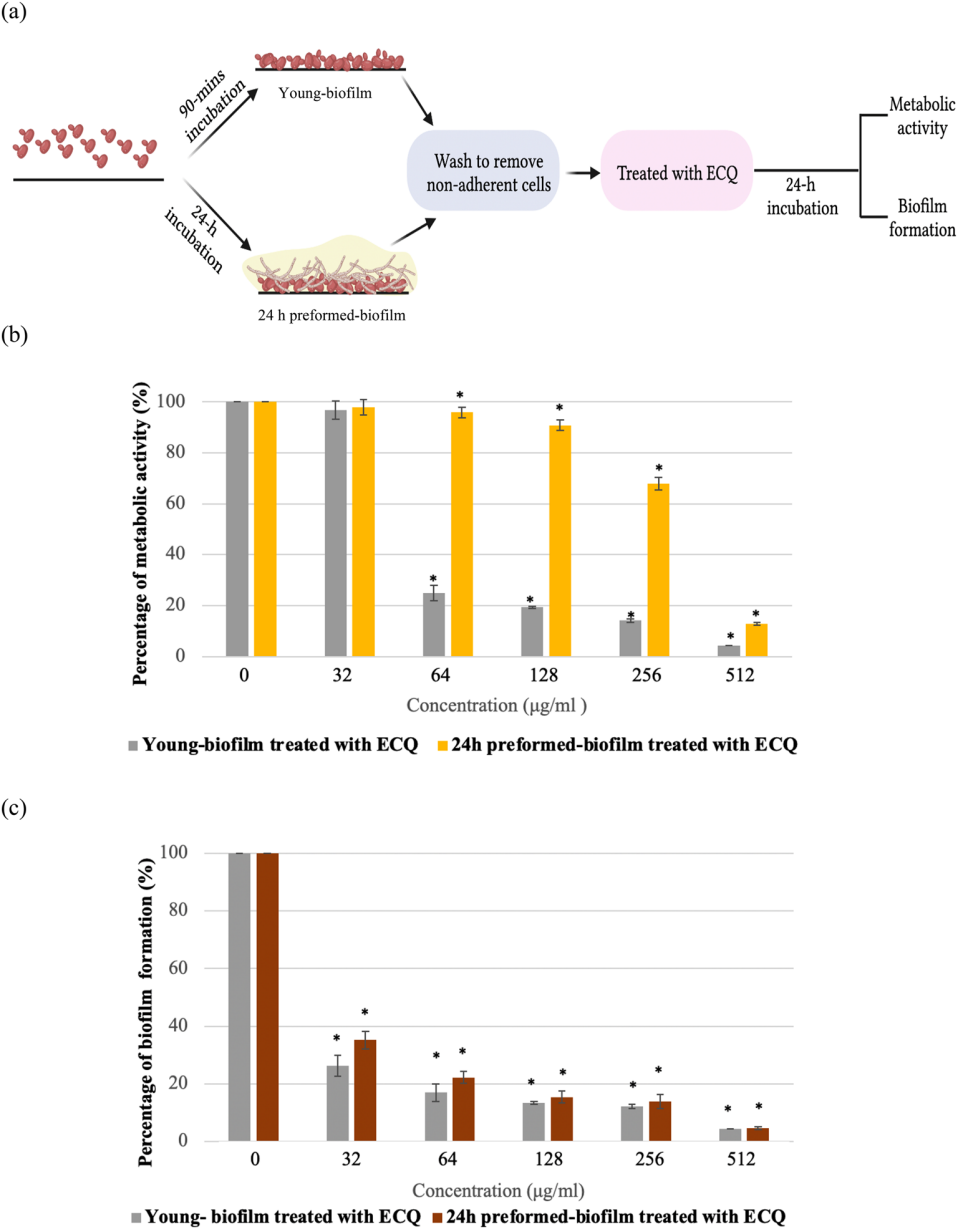

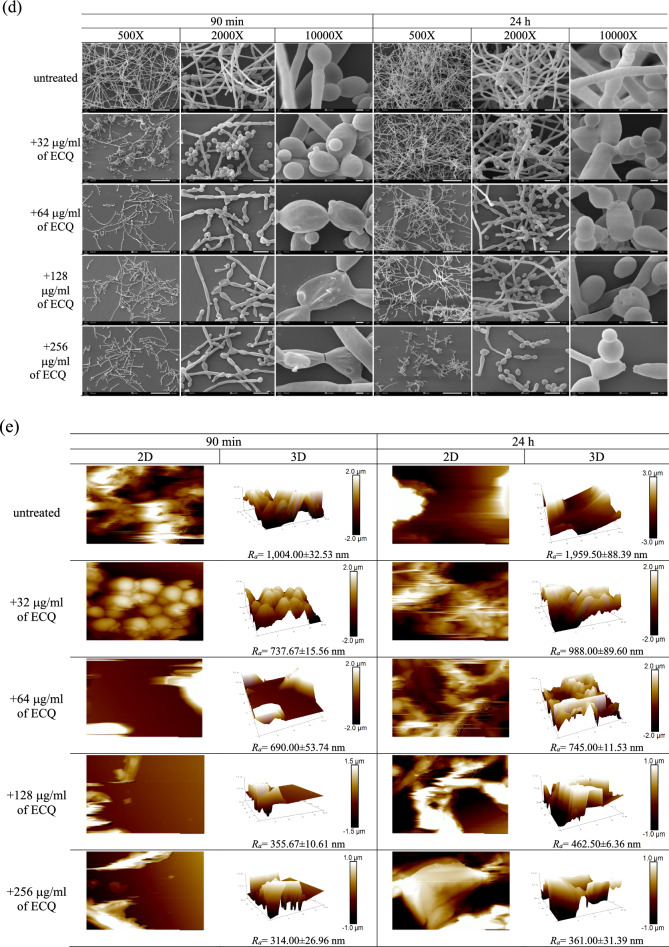
Antibiofilm activity of ECQ against young and 24 h-preformed biofilm of *C. albicans*; (**a**) illustration defines stages of biofilm, (**b**) XTT staining, (**c**) CV staining (**d**) SEM micrographs shown at different magnification (500 × , 2000 × and 10,000 ×) and (**e**) AFM micrographs showing the variation in height of *C. albicans* biofilms. Young biofilm and 24 h-preformed biofilm were formed on coated poly-lysine glass cover slips and treated with ECQ for 24 h. The illustration was created with BioRender. Error bars represent the standard error of the mean (SEM) (*p < 0.05, using one-way ANOVA compared to the untreated condition).

The hyphal form of *C. albicans* is a crucial virulence factor for biofilm formation and maintenance. Next, the ability of ECQ to inhibit *C. albicans* biofilm formation was investigated for the first time. The *C. albicans* biofilms were formed into two stages, young and 24 h-preformed biofilm. The *C. albicans* cells were incubated at 37 °C for 90 min to begin cell adhesion for young biofilms and 24 h for preformed-biofilms as described in Fig. 2a. Utilizing a colorimetric 2,3-bis (2- methoxy-4-nitro-5-sulfophenyl)-5-[(phenylamino)carbonyl]-2H-tetrazolium hydroxide (XTT) reduction test, the effect of ECQ on the metabolic activity of *Candida* biofilms was determined. BIC_80_ (biofilm-inhibiting concentration) is the lowest concentration of ECQ that presented 80% inhibition of biofilm metabolic activity when compared to the untreated condition. In this work, ECQ showed decent ability to inhibit the metabolic activity of *Candida* cells in young biofilm, exhibiting a BIC_80_ of 128 μg/ml. However, ECQ exhibited a higher BIC_80_ (512 μg/ml) against 24 h-preformed biofilm (Fig. 2b) suggesting better protection of cells in 24 h-preformed biofilm compared to the younger one. The ability of ECQ to inhibit biomass of *Candida* biofilm was determined by staining with crystal violet (CV). ECQ showed excellent ability to inhibit biomass of young and 24 h-preformed biofilms at low concentrations: the biomass was reduced by more than 80% starting at concentration of 128 μg/ml for both biofilm stages (Fig. 2c). Therefore, ECQ destroyed biofilms through inhibition of hyphal formation as one key mechanism.

### Structural changes in the hyphal form of *C. albicans* biofilms

To further observe the damaging effect of ECQ on *C. abicans* biofilms, additional experiments were then used to visualize disruption of biofilm structures and alteration of cell surfaces by ECQ. Scanning electron microscopy (SEM) analysis confirmed that ECQ substantially suppressed hyphal and biofilm formation. Young and 24 h-preformed biofilms were created on poly-L-lysine-coated glass cover slips and treated with serial dilution concentrations of ECQ (0–256 g/ml). In untreated young biofilm, cells were mostly hyphal and pseudohyphal forms, while the ECQ-treated biofilm showed shorter hyphae and more pseudohyphae and budding forms (Fig. 2d). Moreover, the effect of ECQ on morphology was observed by cell shrinkage and cell leakage starting from concentrations of 64–256 μg/ml (Fig. 2d). The 24 h-preformed biofilm showed a complex network of biofilm structure which consisted of hyphae and pseudohyphae. The 24 h-preformed biofilm in the presence of 256 μg/ml ECQ showed less hyphal organization and was primarily made up of yeast cells and pseudohyphae (Fig. 2d). The effect of ECQ on the morphology of 24 h-preformed biofilm was smaller than that on young biofilm. No cell shrinkage was found in the treated 24 h-preformed biofilm, while cell leakage started from a concentration of 64–256 μg/ml ECQ in the young biofilm (Fig. 2d).

The effect of ECQ against *C. albicans* hyphae and biofilms was confirmed using the Atomic Force Microscopy** (**AFM). Biofilms were again created on poly-L-lysine-coated glass cover slips in the treatment with ECQ for young and 24 h-preformed biofilm as previously described. The 3D images of AFM provided the clear visualization of *C. albicans* surface on the coated glass cover slips, indicating height and roughness of the biofilm. The roughness of *Candida* biofilms in the presence of ECQ was reduced when compared with untreated biofilms as shown by reduction of the average roughness (*R*_*a*_) (Fig. 2e). The *R*_*a*_ values of untreated young and 24 h-preformed biofilm were 1,004.00 ± 32.53 nm and 1,959.50 ± 88.39 nm, respectively. The *R*_*a*_ values were reduced in a dose-dependent manner after treatment with ECQ. At the highest concentration of 256 μg/ml, the *R*_*a*_ values were reduced by 69% and 82% in young and 24 h-preformed biofilm, respectively, when compared with the untreated condition (Fig. 2e). A variation in the height of biofilms was shown in the Z-axis of the 3-D images, indicating heights of the untreated young and 24 h-preformed biofilms are at least 3 and 2 μm, respectively (Fig. 2e). ECQ also reduced the height of both young and 24 h-preformed biofilm in a dose-dependent manner. At the highest treatment concentration of ECQ at 256 μg/ml, the height of both young and 24 h-preformed biofilms was reduced to less than 1 μm (Fig. 2e). Thus, the AFM provided valuable information on effect of ECQ on surface structure as height and roughness of the biofilms.

This AFM technique has been previously reported to measure adhesion forces of cell surface and also surface elasticity^29^ that are parts of our future plan. Nevertheless, the results obtained from SEM and AFM employed in this current study supported the ability of the ECQ to inhibit hyphal and biofilm formation of *C. albicans* and help to elucidate the mechanisms of action of ECQ. Indeed, the comparative SEM and AFM results provided both simultaneously high-resolution images on fixed *C.albicans* cells as well as rich visualized data with topographical and surface information of yeast cells. The reduction of biofilm complex network observed after ECQ treatments implied morphological and mechanical changes of yeast cell surface and membrane that likely lead to damages of intracellular structures and functions of organelles and other cellular components.

### ECQ destroyed *C. albicans* biofilm by ROS-induced damages of cellular membrane

**Figure 3:**
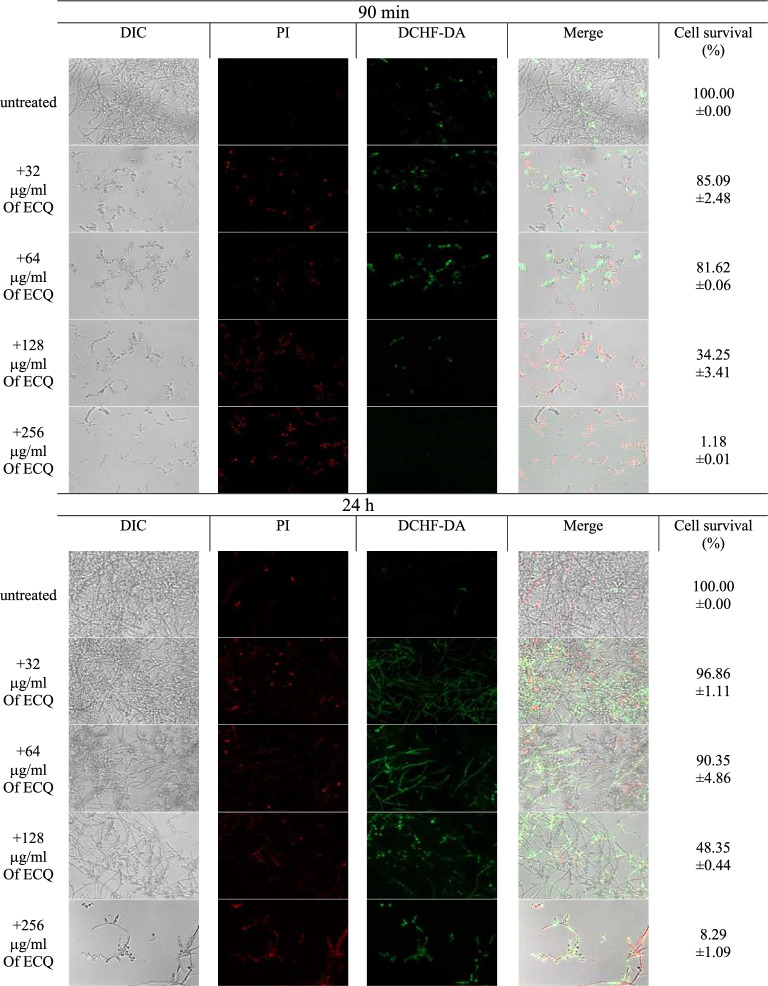
Antifungal ability of ECQ against *C. albicans*. Fluorescence microscopy images of *C. albicans* biofilm cells stained with PI or DCFDA. Error bars represent the standard error of the mean (SEM) (*p < 0.05, using one-way ANOVA compared to the untreated condition). Cell survival (%) represents the percentage of surviving cells relative to untreated cells.

Previously, ECQ has been found to have antifungal activity against *S. cerevisiae* by interfering with actin and lipid homeostasis and impairing the proper function of lipids and structural integrity to the plasma membrane^23^. ECQ has also been reported to induce accumulation of ROSs in the model budding yeast, especially in strains lacking a Pdr-drug transporters to efflux ECQ^24^. The ROSs have been shown to contribute to fungal cell death through the apoptotic effects on different cell types of *C. albicans* by destroying different biological molecules, including nucleic acids, proteins, and lipids^30^. Since, it was the first time to investigate the effect of ECQ on fungal biofilms, the intracellular ROS levels were measured after the ECQ treatments on biofilm-induced *C. albicans* cells. ECQ was found to promote ROS accumulation, observed via 2',7'-Dichlorofluorescin diacetate (DCFDA) at moderate concentrations (32–64 μg/ml) while reducing ROS accumulation at high concentrations (128–256 μg/ml) in the young biofilm (Fig. 3). In contrast, a high number of dead cells observed via propidium iodide (PI) staining was found when concentration of ECQ was increased in young biofilm (Fig. 3). These findings suggested defective membrane structure that was further observed under the microscope. The SEM results displayed the effect of ECQ on morphology: wrinkles started to appear on the cell surface at 64 μg/ml and a more severe effect of cell leakage was observed at high-concentration treatments (128–256 μg/ml) (Fig. 2d). Therefore, ECQ triggered fungal cell death through ROS accumulation in young and old biofilm. Overall results indicated the role of ECQ in biofilm eradication, involving ROS generation, leading to damages of cell wall and/or cell membrane (Figs. 2d and 3).

### Transcriptomic response to ECQ treatment

**Figure 4:**
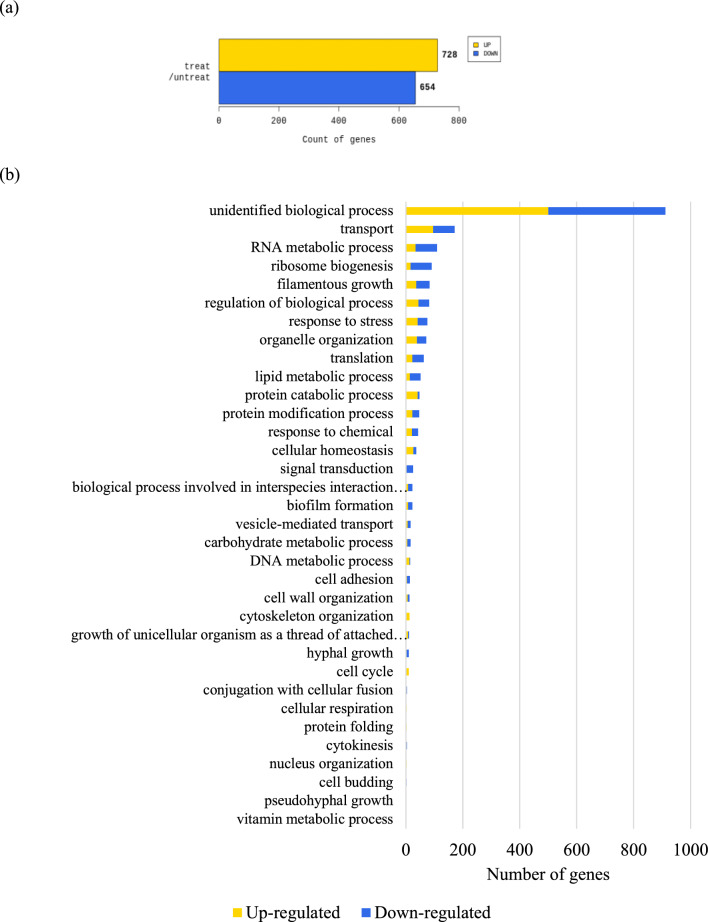

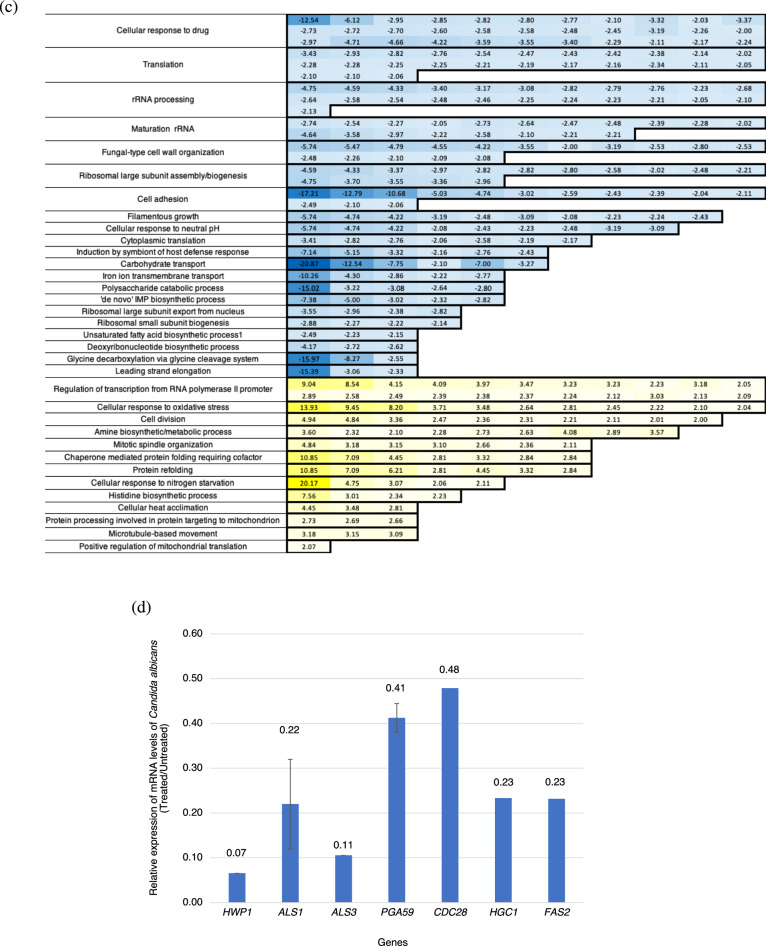
Transcriptomic analysis, GO distribution and qRT-PCR analysis of *C. albicans* genes regulated (> 2-folds) by ECQ treatment; (**a**) transcriptomic provided numbers of genes regulated more than 2-folds by ECQ treatment, (**b**,**c**) the genes were classified GO by biological processes via QuickGO and DAVID bioinformatics resources and (**d**) relative expression of *C. albicans* genes analyzed via qRT-PCR. The *C. albicans* was treated with 128 μg/ml of ECQ for 2 h. The relative mRNA levels of the treated cells were compared to the untreated cells and normalized with a housekeeping gene, *ACT1*. At least two independent qRT-PCR experiments were performed in triplicates.

Then, transcriptomic technology RNA-seq was used to assess the inhibitory effect of ECQ against *C. albicans* cells to better understand its mechanism of action. Transcriptomic analysis showed dramatic changes in expression of over 1,300 genes in various cellular processes (considered significance if expression greater than twofold difference between ECQ-treated versus untreated conditions) (Fig. 4a). Importantly, the expression of many genes involved in filamentous and hyphal growth, cell adhesion, cytoskeleton organization, cell wall synthesis, and biofilm formation were altered in ECQ-treated hyphal induced *C. albicans* cells (Fig. 4b,c). Cell adhesion is an initial step in biofilm formation where *C. albicans* cells adhere to the substratum; in the maturation step, the extracellular matrix is extended to develop the biofilm^31^. The interactions between *C. albicans*, host cells and tissues are tightly controlled by the function of the fungal cell wall. Numerous glycosylated proteins are anchored onto the cell wall via glycosylphosphatidylinositol (GPI) motifs in the outer layer of the cell wall^32^. In this work, ECQ substantially reduced expression of genes involved in GPI anchoring such as *PGA7*, *PGA25, PGA59* and *PGA63* (Table 2 and Supplementary Table S1). The ability of *C. albicans* cells to switch from yeast to hyphal morphology requires hyphal specific genes (HSGs), including *HWP1* encoded for hyphal cell wall protein, *ALS* genes encoded for adhesins^33,34^. We found more than 17-fold downregulation of *HWP1* which encodes an important hyphal cell wall protein that serves in cell-surface adhesion. Once mannosylated, the protein is still attached to the beta-glucan in the cell wall’s outer layer via a remnant of the C-terminal GPI anchor^35,36^.

Adhesins are a family of GPI-linked cell-surface glycoproteins that are encoded by the *ALS* (agglutinin-like sequence) genes and are involved in cell adhesion and adherence to host surfaces^37–39^. In this work, expression of several *ALS* genes, such as *ALS1*, *ALS2*, *ALS3*, *ALS4* and *ALS5* (Table 2 and Supplementary Table S1), was downregulated by ECQ treatment. *UME6*, which is a filament-specific regulator of *C. albicans* hyphal extension and virulence, was also downregulated in ECQ treatments^40^ (Table 2). The *UME6* transcript is normally not detected in yeast cells but is induced upon hyphal induction^41^ and is required for adherence to plastics^42^. The transcription factor Ume6 positively regulates expression of *HGC1* which is hyphae-specific G1 cyclin^43^. Despite not controlling the cell cycle, Hgc1 is important for hyphal morphogenesis^43^. In the present work, we found that ECQ reduced the expression level of *HGC1* along with *CDC28* while increasing expression of *RGA2*, encoding a putative GTPase-activating protein Cdc42 that involved in controlling cell polarity (Table 2). In their interaction, Hgc1 and Cdc28 form a complex regulation by phosphorylation of regulators and element of cell polarity, membrane trafficking, and cell separation, all of which are necessary to maintain hyphal development^44,45^.

### Effect of ECQ treatment on lipid metabolism

In addition of cell surface membrane proteins, the expression of membrane-bound and intracellular lipid-associated enzymes was also largely altered by ECQ treatment (Table 2). In fungi, lipids such as phospholipids, sphingolipids and sterols are important components of plasma membranes. Lipids and lipid‐signaling molecules control cell proliferation and viability which is linked to the virulence of pathogenic fungi^46^. In *S. cerevisiae,* ECQ has been reported to disrupt lipid and sterol organization^23^, which might play a mutual role with actin to reduce virulence. Since ECQ could interfere with *C. albicans* plasma membrane structure (Figs. 2d,e and 3) and disrupt ergosterol homeostasis^23^, we then further analyzed the transcriptomic changes of some genes involved in lipid metabolism. The transcriptomic analysis via RNA-Seq showed that ECQ regulated the expression of genes involved in lipid metabolic processes, including the ergosterol and fatty acid biosynthesis. Expression of genes, involved in ergosterol biosynthesis such as *ERG3*, *ERG4* and *ERG25*, was downregulated while the expression of *UPC2* which is a transcription regulator of ergosterol biosynthesis genes was upregulated (Table 2). Moreover, ECQ reduced the expression of several genes involved in fatty acid biosynthesis, including *FAA4*, *FAD2*, *FAD3*, *FAS1*, *FAS2* and *OLE1* (Table 2). *OLE1* (fatty acid Δ9 desaturase) is essential for viability and is involved in palmitoleic (16:1) and oleic acid (18:1) synthesis^47^. Repression of *OLE1* by genetic repression chemicals results in a decrease of total unsaturated fatty acids and impaired hyphal development^47^. The genes *FAD2* and *FAD3* are orthologs of genes participating in polyunsaturated fatty acid synthesis in *Candida* sp. *FAD2* encodes Δ12-fatty acid desaturase involved in the production of linoleic acid (C18:2) and *FAD3 encodes* omega-3 fatty acid desaturase enzymes involved in the production of alpha-linolenic acid (C18:3) which are major components of membranes^48,49^. Expression of *FAS1* and *FAS2* encoding the beta- and alpha-subunits of fatty acid synthase, respectively, was also affected by treatment with ECQ. It was previously reported that deletion of *FAS2* not only affects fatty acid metabolism but also pathogenicity. The *fas2* homozygous deletion mutant *C. parapsilosis* strain show increased sensitivity to the presence of human serum and is less virulent both in vitro and in vivo^50^.

Expression of some selected genes identified by RNA-Seq were confirmed to be differentially regulated by ECQ, including key genes involved in hyphal growth (*HWP1*), adhesion (*ALS1* and *ALS3*), cell cycle and cell morphology (*CDC28* and *HGC1*) and fatty acid synthesis (*FAS2*), based on the quantitative real-time polymerase chain reaction** (**qRT-PCR) as shown in Fig. 4d. The effect of ECQ on gene expression clearly support the important role of ECQ in the inhibition of hyphal and biofilm as well as lipid biosynthesis.

### Protein–protein interaction network of cell cycle proteins affected by ECQ

**Figure 5:**
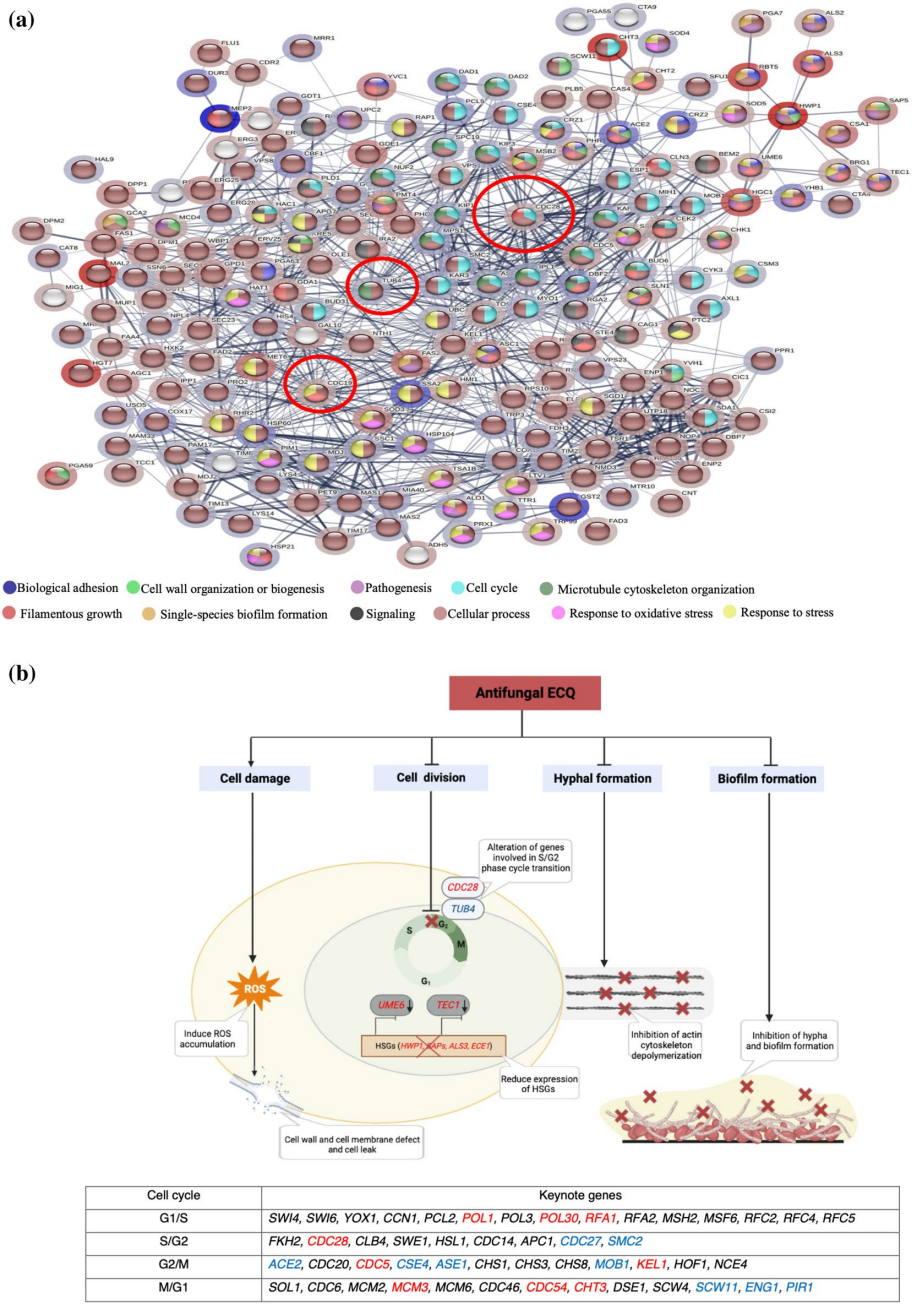
Proposed mechanisms of action of ECQ for inhibition of hyphal and biofilm formation; (**a**) protein–protein interaction network was constructed using the STRING database. In the network, genes serve as nodes and edges serve as protein–protein associations. Line thickness of the network edge indicates the strength of data support. Halo color represents expression of genes from low (red) to high (blue) and (**b**) a model illustration of mechanisms of action of ECQ. Periodic expression of key note genes for each cell cycle were identified by Côte et al.^52^ ECQ dependent keynote genes were labeled as red letter (down-regulated expression), blue letter (up-regulated expression) and black letter (unchanged expression).

After, using a computer-aided software, protein–protein interaction analysis of *Candida* cells was conducted to identify the intertwined relationships of these ECQ-dependent genes. Protein–protein interactions of genes of interest identified from the RNA-seq results were presented (Fig. 5a). The hubs were analyzed by gene ontology (GO) and were mostly classified under cellular processes, cell cycle process, filamentous growth, microtubule cytoskeleton organization, response to stress and oxidative stress. Cdc28 showed the highest node degree of protein–protein interaction (39 interactions) which suggests an interesting effect of ECQ (Fig. 5a). Cdc28p, a cyclin-dependent protein kinase involved in determination of cell morphology during the cell cycle and peaks at S/G2 phase, has been reported to exhibit filamentous growth and abnormal hyphal morphology in repressible mutants^51,52^. Tub4, a gamma-tubulin protein involved in microtubule nucleation^53^, also presented a high node degree (38 interactions) (Fig. 5a). *TUB4* expression is induced during adherence to polystyrene and cell-cycle S/G2 phase^52,54^. In addition, Cdc19 also showed a high node degree of interaction with other ECQ targets (37 interactions) (Fig. 5a). *CDC19* encodes a putative pyruvate kinase which is found on the cell surface of the yeast form of *C. albicans*^55^. Cdc19p is also strongly downregulated in hyphal form of another yeast *Yarrowia lipolytica*^56^. Downregulation of *CDC28* and *CDC19* along with upregulation of *TUB4* expression might be a primary response of *C. albicans* cells to the interfering effect of ECQ on hyphae and filamentous growth during the cell cycle, providing a molecular link to coordinated expression of genes in other connected pathways during cell transition (Table 2, Figs. 4 and 5a).

As an actin inhibitor, ECQ showed potent antifungal activity against *C. albicans* and reduced *C. albicans* metabolic activity. The mechanism of action of ECQ mediated antibiofilm activity against the young and 24 h-preformed biofilms and inhibition of hyphal formation may be primarily triggered through cell cycle and cytoskeleton organization. It has been previously reported that F-actin plays a role as a regulatory agent of hyphae-specific gene expression at the bud–hyphae transition^57^. Moreover, additional cytochalasin compounds have been reported to inhibit hyphal formation. For example, Cytochalasin A, which destabilizes actin filaments, and latrunculin A, which sequesters actin monomers, are reported to inhibit hyphae-specific adhesin gene *HWP1* gene expression and block hyphal formation^57^. Similar effect is observed in ECQ in *C. albicans* (Fig. 1 and Table 2). Alteration of lipid composition^23^ and microenvironment following ECQ treatment might effect hyphal tip development and cell division process, leading to alteration in cellular morphology. Actin filaments play a critical role in sterol polarization and hyphal morphogenesis since they direct secretory vesicles to the hyphal tip and are damaged by latrunculin^58^. In fact, this protruding area exhibits more intense staining with the binding agent such as filipin, indicating a change in ergosterol levels or an increased accessibility of filipin ^58^. The presence of actin patches in this subapical region has been shown to facilitate the endocytosis to support tip growth. ECQ exhibited effect on transcription level of *CDC28* and *TUB4,* both genes involved in cell cycle at S/G2 transition phase. Putative hyphal genes in *C. albicans* were identified by their expression at peak levels during the S/G2 transition, and their promoters are particularly abundant in Tec1 DNA binding site motifs, which are known to play crucial roles in hyphal growth^59^. Importantly, *TEC1* expression was also downregulated in ECQ treatments (Table 2 and Fig. 5b). Overall, the results suggested the pivotal role of ECQ in the inhibition of yeast to hyphal transition thereby, preventing the formation of *Candida* biofilms.

To summarize, we present a model of the poorly known antifungal agent ECQ following characterization of its mode of action in antihyphal and antibiofilm against *C. albicans* (Fig. 5b). Using the transcriptomic data of ECQ-treated *C. albicans*, we provide key evidences to support the connection between the cell cycle-dependent ECQ-targeted actin depolymerization to disruption of hyphal and filamentous growth which eventually results in ROS-mediated cell death and reduction of biofilm mass. Since biofilms produced by fungal pathogens are intrinsically resistant to the current clinically-available antifungal agents and essential for pathogenesis of catheter infections in the hospital settings, identification of novel antibiofilm agents such as ECQ may provide an avenue for antifungal drug development to effectively target key virulent factors. Further work is underway to formulate ECQ to improve its antifungal efficacy against virulent pathogens’ biofilms.

## Materials and methods

### Effect of ECQ on hyphal formation

The effect of ECQ on hyphal formation was performed as described by Haque et al.^60^ with modifications. In this experiment, RPMI-1640 medium (R6504, Sigma) containing 10% FBS (Invitrogen) was used as the culture medium. A *Candida* cell suspension was prepared at 1 × 10^6^ CFU/ml in the culture medium containing different concentrations of ECQ (0, 32, 64, 128 and 256 μg/ml). The cell suspension was incubated at 200 rpm at 37 °C for different periods (0, 1, 2 and 24 h). Samples were collected and washed twice with phosphate-buffered saline (PBS) and observed under a bright field using a 40 × objective lens. Hyphal cells were counted and normalized with total cells as a percentage.

### Combination of ECQ and sophorolipids on hyphal formation

A *Candida* cell suspension was prepared at 1 × 10^6^ CFU/ml in RPMI-1640 medium (R6504, Sigma). The cells were treated with combination of various concentrations of ECQ (0, 32, 64 μg/ml) and sophorolipids (0, 8, 16 μg/ml). The cell suspension was incubated at 200 rpm at 37 °C for 5 h. Samples were collected and washed twice with phosphate-buffered saline (PBS) and observed under a bright field using a 40 × objective lens.

### Assay for in vitro biofilm formation

Flat-bottomed 96-well microplates were used to form *C. albicans* ATCC90028 biofilm as described by Stepanović et al.^61^ with some modifications^62^. RPMI-1640 medium with l-glutamine without sodium bicarbonate (Sigma) (buffered with 0.165 M morpholinepropanesulfonic acid to a pH of 7) was used as a culture medium. 1 × 10^6^ CFU/ml of cell suspension in the RPMI-1640 medium was prepared and 200 µl of the cell suspension was added into the 96-well microplates. The plates were then incubated at 37 °C without shaking for 90 min to begin cell adhesion for young biofilms and 24 h for 24 h-preformed-biofilms. Non-adherent cells were removed after the incubation period was completed by rinsing the wells in sterile PBS (pH 7.2, 0.15 M). Each well received 200 µl of freshly prepared RPMI-1640 that was serially diluted with ECQ before being cultured for 24 h at 37 °C. The culture broth was gently removed, and each well was rinsed twice with PBS and allowed to air-dry for 30 min. In order to quantify biofilm formation, 100 µl of a 1% (w/v) CV solution was added to stain the biofilm and further incubated for 30 min. To remove any excess of CV, the plate was washed with PBS three times and the plate allowed to dry. After that, 200 µl of absolute ethanol was added to release the dye from the biofilm. Then, a new 96-well microplate was prepared and 100 µl of solution was transferred to the new plate. A Multiskan Sky microplate reader (Thermo Scientific, USA) was used to measure the absorbance at 590 nm.

### Assay for measuring metabolic activity of *Candida* biofilms

The metabolic activity of *Candida* biofilms was determined as described by Ramage et al.^63^.with modifications. XTT was used; the assay relies on converting the XTT to formazan product by mitochondrial dehydrogenase. The working solution had a final concentration of 1 μM of freshly prepared menadione and 0.5 g/l of XTT in PBS. *Candida* biofilm was prepared as described above and 200 μl of the working solution was added into the 96-well plate. The plate was further incubated in the dark at 37 °C for 1 h. To measure the colorimetric change in the XTT reduction, a Multiskan Sky microplate reader (Thermo Scientific, USA) was used and detected at 490 nm.

### Qualitative analysis of *C. albicans* biofilm by SEM

SEM was used to examine the qualitative impact of ECQ on biofilms. The experiment was conducted as previously described by Dong et al.^64^. Poly-l-lysine solution (0.1% wt/vol) was used to coat glass cover slips. To sterilize the coated glass cover slips, UV radiation was used for 1 h while the cover slips were circulated in laminar air. The cover slips were then transferred to 6-well plates (Nunc) for formation of biofilms. A *Candida* cell suspension was prepared at 1 × 10^6^ CFU/ml in 2 ml of RPMI-1640 medium and added into the well plates containing coated glass cover slips. The well plates were then incubated at 37 °C for 90 min to begin cell adhesion for young biofilms and 24 h for 24 h-preformed biofilms. Non-adherent cells were eliminated after the incubation period was complete by rinsing the wells in sterile PBS. Each well received 2 ml of freshly prepared RPMI-1640 that was serially diluted with ECQ before being cultured for 24 h at 37 °C. After the incubation period was complete, cover slips were moved to fresh 6-well plates and washed with PBS three times. Then formaldehyde (4% vol/vol) was used to fix the biofilms overnight. Coverslips were washed for 10 min twice with PBS followed by once with deionized water. The dehydration step was performed by increasing the concentration of ethanol starting from 30 to 50%, 70%, 95% and 100% for 10 min and repeating three times with 100% ethanol. After that, the biofilms were put into a critical point dryer (Leica model EM CPD300, Austria) for drying and coated with gold (sputter coater, Balzers model SCD 040, Germany). The biofilms were visualized using a SEM and energy dispersive X-ray spectrometer—SEM–EDS (Jeol JSM-IT500HR).

### AFM

Young and 24 h-preformed biofilms were formed as previously described in the SEM analysis section. Samples were analyzed by AFM (Bruker Instruments). All images were collected in tapping mode using a RFESPA-75 AFM probe with a spring constant of 2.8 N/m.

### DCFDA and PI evaluation

DCFDA and PI evaluation was conducted as reported by Gupta et al.^65^ with modifications. In brief, young and 24 h-preformed biofilms were formed as previously described. After treatment with ECQ, the samples were eliminated by gently washing the wells with PBS. Then the samples were incubated for 20 min at room temperature with 5 μg/ml of PI (Sigma-Aldrich) and 0.1 mM of DCFDA (Sigma-Aldrich) in the dark. After incubation, the samples were washed with PBS and observed using a ZEISS Apotome.2 fluorescent microscope (ZEISS, Germany). The fluorescence of DCFDA was evaluated at 485 nm excitation and 520 nm emission wavelengths, while the fluorescence of PI was evaluated at 543 nm excitation and 617 nm emission wavelengths.

### Transcriptomic analysis and qRT-PCR

Transcriptomic analysis was performed using RNA-seq analysis. *C. albicans* cell suspension was prepared at an optical density (OD_600_) of 0.5 to be the cell starter in RPMI-1640 medium containing 10% FBS (Invitrogen). The cell suspension was treated with ECQ at 128 μg/ml at 37 °C with 200 rpm agitation for 2 h. After incubation, samples were washed twice with 0.1% diethylpyrocarbonate (DEPC)-treated water. Total RNAs were collected as reported by Schmitt et al.^66^ DNA contamination was eliminated using DNase. The RNAs were purified using a Qiagen RNeasy Mini Kit and the quality checked by agarose gel electrophoresis. For RNA-seq analysis, an RNA library was constructed using a TruSeq RNA Sample Prep Kit v2 (Illumina, Inc., USA). The Excel-Based Differentially Expressed Gene Analysis (ExDEGA) program was used to perform DEG (differentially expressed gene) analysis on a comparison pair (Treated with ECQ vs Untreated). QuickGO (www.ebi.ac.uk/QuickGO/) and DAVID bioinformatics resources (https://david.ncifcrf.gov/) were used to analyze GO and biological processes were categorized. For qRT-PCR analysis, cDNA was synthesized by using the qPCRBIO cDNA synthesis kit (PCRBIOSYSTEMS, UK). Luna® Universal qPCR Master Mix (NEB) was used for the reaction mixtures. The parameters used for qRT-PCR were previously described by Haque^60^. Gene-specific oligonucleotides and housekeeping internal control (*ACT1*) genes were shown (Table 1).

**Table 1:** Gene-specific oligonucleotides.

Primer	Sequence	References
PGA59-F	AAGTCACTACTGGTGTCACC	This study
PGA59-R	CAGCAGTAGAAACTGGTGG	This study
CDC28-F	GTACCGCCATTAGAGAAATCTCG	This study
CDC28-R	CTAGTCCAACTCCTTGAGGAATAC	This study
HGC1-F	CCAATAGTATCATTGGCGGG	This study
HGC1-R	GATACTGGAGTAGTAGAGCCAG	This study
FAS2-F	GCTGGTATGGCCAATAGAAC	This study
FAS2-R	CTGCTGGATCTGGCTTGTAG	This study
CaALS1-RTS	AGCTGTTGCCAGTGCTTC	Haque et al.^53^
CaALS1-RTAS	AATGTGTTGGTTGAAGGTGAG	Haque et al.^53^
CaALS3-RTS	CAACATCAACCAACCAATCTC	Haque et al.^53^
CaALS3-RTAS	TGAATAACAGAACCAGATCCG	Haque et al.^53^
CaACT1-RTS	GGTTTGGAAGCTGCTGGTATTGACC	Haque et al.^53^
CaACT1-RTAS	ACGTTCAGCAATACCTGGGAACATG	Haque et al.^53^

### Protein–protein interaction network analysis

Protein–protein interactions of genes of interest from the RNA-seq analysis, including genes presented in Table 2 were further evaluated using the STRING database as previously described^67^. In the network represented, genes serve as nodes and edges serve as protein–protein associations. The line thickness of the network edge indicates the strength of data support. The interaction score was considered to have minimum confidence at 0.400.

**Table 2:** Gene ontology (GO) distribution of interested *C. albicans* genes regulated (> twofold) by ECQ.

Biological processes	Genes and expression level													
Filamentous growth	***MAL2***	***HGC1***	***PGA59***	***ASC1***	***MSB2***	***PMT4***	***RNR1***	***RFX2***	***CAS4***	***GDA1***	***CHT2***	***FET34***	***ERG3***	***NOC2***
	***MTS1***	***YVH1***	***CSI2***	***KRE5***	***ALS1***	***GAL10***	***LHP1***	***TCC1***	***CDC19***	***RPS10***	***SLN1***	***KEL1***	***CDC28***	***ENP1***
	***BEM2***	***TEC1***	***PLD1***	***CDC5***	*RTA4*	*RHB1*	*MRP2*	*STE23*	*SSN6*	*HAL9*	*CTA9*	*VPS23*	*RAP1*	*PPR1*
	*CTA4*	*GCN4*	*KAR3*	*ALO1*	*CAT8*	*BUD6*	*LYS14*	*FGR39*	*CRZ1*	*PCL5*	*FCR1*	*YHB1*	*RBF1*	
Organelle organization	***FAV1***	***CSP2***	***FLU1***	***ELF1***	***UTP18***	***NOP4***	***DBP7***	***SDA1***	***LTV1***	***HMI1***	***ENP2***	***NOC2***	***CIC1***	***MDJ2***
	***TSR1***	***VPS13***	***BUD22***	***FAA4***	***SEC12***	***SGD1***	***RPA135***	***HAC1***	***NTH1***	*CSM3*	*ESP1*	*UBC4*	*MTR10*	*RTA4*
	*MAM33*	*VPS8*	*PTR3*	*PRO2*	*RAP1*	*PPR1*	*CAT8*	*MAS2*	*CCE1*	*AXL1*	*LYS4*	*MAS1*	*SSC1*	*HAP42*
	*PUT4*	*KAR9*	*MDJ1*	*ZCF8*	*TRP3*	*CSE4*	*MRR1*	*COX17*	*ZCF19*	*HSP60*				
Lipid metabolic process	***HGT7***	***FAS2***	***GDE1***	***DPP1***	***DPM1***	***FAS1***	***PMT4***	***ERG25***	***PGA38***	***IPP1***	***ERG3***	***WBP1***	***HXK2***	***PGA48***
	***MCD4***	***RNR21***	***FAD2***	***CNT***	***OST1***	***FAA4***	***OLE1***	***FAD3***	***DPM2***	***ERG4***	***PLB5***	***RHD1***	***CYK3***	***SCW11***
	*ERG28*	*BMT5*	*COX15*	*ALK8*	*UPC2*	*EBP1*	*ESP1*	*AXL1*	*SAP3*	*ZCF19*				
Transport	***RBT5***	***AGC1***	***FLU1***	***ERV25***	***PET9***	***SDA1***	***CDR2***	***MDJ2***	***CNT***	***NMD3***	***SEC23***	***TIM17***	***MUP1***	***FAA4***
	***PGA63***	***SEC26***	*UBC4*	*TIM8*	*MTR10*	*PAM17*	*MAM33*	MAM33	*NPL4*	*APG7*	*PRO2*	*TIM13*	*TIM22*	*LYS4*
	*MAS1*	*SSC1*	*MIA40*	*OPT4*	*COX17*	*HSP60*	*DUR3*							
Response to stress	***MET6***	***MSB2***	***RFX2***	***PHO84***	***TSA1B***	***CHK1***	***SOD5***	***GAL10***	***SLN1***	***SSK1***	*PIM1*	*TTR1*	*PRX1*	*FDH3*
	*PST3*	*SOD4*	*ALO1*	*HSP21*	*MPS1*	*HSP104*	*HSP60*	*YHB1*	*CIP1*	*GST2*				
Response to chemical	***SOD3***	***FET34***	***PTC2***	***MIG1***	***CDR2***	***CNT***	***HAC1***	***TRP99***	*HAT1*	*FDH3*	*ALO1*	*HAP3*	*MPS1*	*YHB1*
	*CIP1*	*SSA2*												
Biofilm formation	***RBT5***	***CSA1***	***SAP5***	***PMT4***	***GCA2***	***ADH5***	***CAS4***	***RHR2***	***ALS1***	***PGA7***	*SOH1*	*GCN4*	*MET4*	*ZCF8*
	*IFD6*	*CRZ2*												
Signal transduction	***ASC1***	***RAS1***	***PHO84***	***GIN1***	***CDC5***	***STE4***	***PDE1***	***CEK2***	***SLN1***	***HAC1***	***CAG1***	***SSK1***	*CRZ1*	*MPS1*
	*RGA2*	*REP1*												
Cell adhesion	***HWP1***	***RBT5***	***ALS3***	***YVC1***	***ASC1***	***GPD1***	***TRY6***	***IRA2***	***RHR2***	***BRG1***	***ALS4***	***ALS1***	***UME6***	***PHR1***
	***TOM1***	***PGA25***	***PGA63***	***ALS2***	***SSK1***	***ALS5***	***TEC1***	*MIH1*	*HIS4*	*HWP2*	*ACE2*	*PGA55*	*CRZ2*	*MEP2*
	*PGA23*	*CRZ1*	*PGA28*	*SFU1*										
Cell wall organization	***CSP2***	***MSB2***	***PHR3***	***KRE5***	***HAC1***	*RLM1*	*CEK1*	*PHR2*						
Hyphal growth	***HAC1***	***PHO84***	***ALS4***	***CDC28***	***CFL11***	***CLN3***	***ALS1***	*GDT1*	*DBF2*	*CRZ1*	*RGA2*			
Cytoskeleton organization	***CDC28***	***CDC5***	*DAD1*	*KAR3*	*KIP3*	*ASE1*	*TUB4*	*MYO1*	*KIP1*	*MPS1*	*DBF2*	*SPC19*	*IPL1*	*DAD2*
	*NUF2*	*NPL4*	*KAR9*											
Growth of unicellular organism as a thread of attached cells	***GAL10***	***STE4***	***CEK2***	***ASC1***	***YVC1***	*RAP1*	*USO5*	*CTA4*	*HAL9*	*SOH1*	*MRR1*	*CAT8*	*WOR2*	
Cell cycle	***CDC28***	***CHT3***	***CLN3***	*ESP1*	*DAD1*	*KIP3*	*KIP1*	*MPS1*	*DBF2*	*CSE4*	*SPC19*	*IPL1*	*DAD2*	*CBF1*
	*CRZ1*	*BUD31*												
Conjugation with cellular fusion	***PLD1***	***STE4***	***CEK2***	***CAG1***	*KAR3*	*USO5*								
Cytokinesis	***MSB2***	***CHT3***	*MYO1*	*CRZ1*	*MOB1*	*BUD31*								
Nucleus organization	***FAS2***	***CAG1***	*SMC2*	*KAR3*	*USO5*									
Cell budding	***CLN3***	*CRZ1*												
Pseudohyphal growth	*RAP1*													

The genes were analyzed with https://www.ebi.ac.uk/QuickGO and classified by biological processes and labeled as bold (down-regulated expression) and underlined (up-regulated expression).

### Statistical analysis

The results were expressed as means ± standard deviations. SPSS (Statistical Package for the Social Sciences) Statistics 26.0 software (IBM, NY, USA) was used for statistical analysis and one-way ANOVA was performed followed by Tukey’s pairwise comparison. A *p*-value of < 0.05 was regarded as significant. A minimum of three independent tests were conducted in triplicate.

## Supplementary Information

Supplementary Tables.

## Data Availability

Raw data reported in this paper have been deposited at the interactive web-based site of the Excellent Research Laboratory for Yeast Innovation (Bangkok, Thailand) under the Project ID: ECQ0001 at https://drive.google.com/drive/folders/1BVOErQLlTUSvsf15BVm_huF3eaFDYukm.
