# Supplementary material for: Inhibition of cell cycle-dependent hyphal and biofilm formation by a novel cytochalasin 19,20‑epoxycytochalasin Q in Candida albicans
Source: Sci Rep. 2023 Jun 15;13:9724. doi: 10.1038/s41598-023-36191-4 (PMC10272203; doi:10.1038/s41598-023-36191-4)
Supplement: Supplementary file 1 — Supplementary Tables. [file 41598_2023_36191_MOESM1_ESM.pdf]

Table S1 Gene ontology (GO) distribution of down- regulated genes by ECQ. The genes were analyzed with https://david.ncifcrf.gov and classified by biological processes.

Biological processes	Genes and expression level									
Cellular response to drug	*HGT7* (-12.54)	*GDE1* (-6.12)	*FAS1* (-4.71)	*CAR2* (-4.66)	*PMT4* (-4.22)	*UTP5* (-3.59)	*RRS1* (-3.55)	*NOP4* (-3.40)	*RLP24* (-3.37)	*IMH3* (-3.32)
	CAS4 (-3.19)	*RPF2* (-2.97)	*UTP20* (-2.95)	*ENP2* (-2.85)	*MRT4* (-2.82)	*NOC2* (-2.80)	*ROA1* (-2.77)	*MIG1* (-2.73)	*CLN3* (-2.72)	*CDR2* (-2.70)
	DRS1 (-2.60)	*DRS1* (-2.60)	*UTP15* (-2.58)	*NOP14* (-2.58)	*KRE5* (-2.48)	*CNT* (-2.45)	*RRP9* (-2.29)	*GAL102* (-2.26)	*PMT2* (-2.24)	*GAR1* (-2.17)
	EFT2 (-2.11)	*DIP2* (-2.10)	*KSP1* (-2.03)	*PRS1* (-2.00)						
Translation	*RPL18* (-3.43)	*RPS20* (-2.93)	*RPL5* (-2.82)	*SSB1* (-2.76)	*RPL4B* (-2.54)	*RPL10A* (-2.47)	*RPS42* (-2.43)	*TMA19* (-2.42)	*RPS8A* (-2.38)	*RPP2A* (-2.34)
	RPL13 (-2.28)	*RPS26A* (-2.28)	*RPS21B* (-2.25)	*RPS21* (-2.25)	*RPL35* (-2.21)	*RPP1B* (-2.19)	*RPL43A* (-2.17)	*RPL12* (-2.16)	*RPL23A* (-2.14)	*RPL28* (-2.11)
	RPS12 (-2.10)	*RPS17B* (-2.10)	*YST1* (-2.06)	*RPP2B* (-2.05)	*RPL25* (-2.02)					
rRNA processing	*NSA1* (-4.75)	*MDN1* (-4.58)	*SSF1* (-4.33)	*DBP2* (-3.40)	*DBP7* (-3.17)	*DBP3* (-3.07)	*MRT4* (-2.82)	*KRR1* (-2.79)	*SSB1* (-2.76)	*UTP21* (-2.68)
	BUD21 (-2.64)	*UTP15* (-2.57)	*RPF1* (-2.53)	*CSI2* (-2.47)	*TSR1* (-2.45)	*RPL3* (-2.24)	*POP3* (-2.23)	*PWP1* (-2.23)	*IMP4* (-2.22)	*DBP8* (-2.21)
	HCA4 (-2.12)	*SPB4* (-2.10)	*ENP1* (-2.04)							
Fungal-type cell wall organization	*PGA59* (-5.74)	*SAP5* (-5.47)	*CSP2* (-4.79)	*MSB2* (-4.55)	*PMT4* (-4.22)	*PHR3* (-3.55)	*CAS4* (-3.19)	*SUN41* (-2.80)	*TSA1B* (-2.53)	*TSA1* (-2.53)
	KRE5 (-2.48)	*PHR1* (-2.26)	*PRS5* (-2.10)	*HAC1* (-2.09)	*SSK1* (-2.08)	*PRS1* (-2.00)				
Ribosomal large subunit assembly	*MDN1* (-4.59)	*SSF1* (-4.33)	*RLP24* (-3.37)	*RPF2* (-2.97)	*MRT4* (-2.82)	*RPL5* (-2.82)	*RPP0* (-2.58)	*CSI2* (-2.48)	*RPL3* (-2.21)	*RPL25* (-2.02)
Filamentous growth of a population of unicellular organisms in response to neutral pH	*PGA59* (-5.74)	*ASC1* (-4.74)	*PMT4* (-4.22)	*CAS4* (-3.19)	*GDA1* (-3.09)	*KRE5* (-2.48)	*ALS1* (-2.43)	*PMT2* (-2.24)	*OLE1* (-2.23)	*SSK1* (-2.08)
Cell adhesion	*HWP1* (-17.21)	*INT1* (-5.03)	*ASC1* (-4.74)	*TRY6* (-3.02)	*BRG1* (-2.59)	*ALS1* (-2.43)	*UME6* (-2.39)	*PGA63* (-2.11)	*TEC1* (-2.04)	
Cellular response to neutral pH	*PGA59* (-5.74)	*ASC1* (-4.74)	*PMT4* (-4.22)	*CAS4* (-3.19)	*GDA1* (-3.09)	*KRE5* (-2.48)	*ALS1* (-2.43)	*OLE1* (-2.23)	*SSK1* (-2.08)	
Maturation of LSU-rRNA	*MAK16* (-2.74)	*CIC1* (-2.73)	*RPL8B* (-2.64)	*RPF1* (-2.54)	*RPL10A* (-2.47)	*RRP15* (-2.27)	*NSA2* (-2.05)	*RPL82* (-2.02)		
Maturation of rRNA from tricistronic rRNA transcript (SSU-rRNA, 5.8S rRNA, LSU-rRNA)	*HIT1* (-4.64)	*YTM1* (-3.58)	*RPF2* (-2.97)	*MAK16* (-2.74)	*HAS1* (-2.22)	*ERB1* (-2.21)	*RPL35* (-2.21)			
Ribosomal large subunit biogenesis	*NSA1* (-4.75)	*NIP7* (-3.70)	*RRS1* (-3.55)	*REI1* (-3.36)	*SDA1* (-2.96)	*MRT4* (-2.82)	*NOC2* (-2.80)			
Cytoplasmic translation	*RPL9B* (-3.41)	*RPL15A* (-2.82)	*SSB1* (-2.76)	*RPP0* (-2.58)	*RPP1B* (-2.19)	*RPL2* (-2.17)	*YST1* (-2.06)			
Maturation of SSU-rRNA	*LSM6* (-2.73)	*NOP14* -2.58)	*CHR1* (--2.48)	*HBR3* (-2.39)	*BUD22* (-2.28)	*DIP2* (-2.10)				
Cell adhesion involved in single-species biofilm formation	*RBT5* (-12.79)	*ALS3* (-10.68)	*ALS4* (-2.49)	*ALS1* (-2.43)	*ALS2* (-2.10)	*ALS5* (-2.06)				
Induction by symbiont of host defense response	*MET6* (-7.14)	*ENO1* (-5.15)	*IMH3* (-3.32)	*SSB1* (-2.76)	*ALS1* (-2.43)	*CDC19* (-2.16)				
Carbohydrate transport	*HGT9* (-20.87)	*HGT7* (-12.54)	*HGT2* (-7.75)	*MAL31* (-7.00)	*HUT1* (-3.27)	*HGT14* (-2.10)				
Cell adhesion involved in multi-species biofilm formation	*ALS3* (-10.68)	*ALS4* (-2.49)	*ALS1* (-2.43)	*ALS2* (-2.10)	*ALS5* (-2.06)					
Cell-cell adhesion	*ALS3* (-10.68)	*ALS4* (-2.49)	*ALS1* (-2.43)	*ALS2* (-2.10)	*ALS5* (-2.06)					
Polysaccharide catabolic process	*CHT3* (-15.02)	*GCA1* (-3.22)	*CHT2* (-3.08)	*SUN41* (-2.80)	*SIM1* (-2.64)					
'de novo' IMP biosynthetic process	*ADE13* (-7.38)	*ADE6* (-5.00)	*ADE8* (-3.02)	*ADE2* (-2.82)	*ADE4* (-2.32)					
Iron ion transmembrane transport	*FRP1* (-10.26)	*CTR1* (-4.30)	*FET34* (-2.86)	*FTR1* (-2.77)	*FET3* (-2.22)					

Ribosomal large subunit export from nucleus	*RRS1* (-3.55)	*SDA1* (-2.96)	*MRT4* (-2.82)	*NMD3* (-2.38)
Ribosomal small subunit biogenesis	*LTV1* (-2.88)	*NOP6* (-2.27)	*HAS1* (-2.22)	*SGD1* (-2.14)
Maturation of 5.8S rRNA	*MAK16* (-2.74)	*RPF1* (-2.54)	*RRP15* (-2.27)	*NSA2* (-2.05)
Deoxyribonucleotide biosynthetic process	*RNR1* (-4.17)	*RNR3* (-2.72)	*RNR21* (-2.62)	
Leading strand elongation	*POL1* (-15.39)	*POL30* (-3.06)	*POL2* (-2.33)	
Unsaturated fatty acid biosynthetic process1	*FAD2* (-2.49)	*OLE1* (-2.23)	*FAD3* (-2.15)	
Glycine decarboxylation via glycine cleavage system	*GCV2* (-15.97)	*GCV1* (-8.27)	*GCV3* (-2.55)	

Table S2 Gene ontology (GO) distribution of up- regulated genes by ECQ. The genes were analyzed with https://david.ncifcrf.gov and classified by biological processes.

**Biological processes**	**Genes and expression level**									
Regulation of transcription from RNA polymerase II promoter	*CRZ2* (9.04)<br>*CTA8* (2.89)<br>*MED7* (2.12)	*ACE2* (8.54)<br>*SOH1* (2.58)<br>*TFB3* (2.09)	*STP1* (4.15)<br>*UPC2* (2.49)<br>*RLM1* (2.05)	*CRZ1* (4.09)<br>*ADR1* (2.39)	*GAT1* (3.97)<br>*SKO1* (2.38)	*STP4* (3.47)<br>*SFU1* (2.37)	*MED8* (3.23)<br>*SFU1* (2.37)	*HAP3* (3.23)<br>*REP1* (2.24)	*MET4* (3.18)<br>*RPN4* (2.23)	*GCN4* (3.03)<br>*MET28* (2.13)
Cellular response to oxidative stress	*GST2* (13.93)<br>*CAP1* (2.04)	*CIP1* (9.45)	*AHP1* (8.20)	*MPS1* (3.71)	*HSP21* (3.48)	*PST3* (2.81)	*FDH3* (2.64)	*PRX1* (2.45)	*TTR1* (2.22)	*PIM1* (2.10)
Cell division	*DBF2* (4.94)	*DAD1* (4.84)	*SMC2* (3.36)	*CEK1* (2.47)	*NUF2* (2.36)	*BRN1* (2.31)	*SPC19* (2.21)	*DAD2* (2.11)	*MIH1* (2.01)	*CDC27* (2.00)
Chaperone mediated protein folding requiring cofactor	*SSA2* (10.85)	*HSP70* (7.09)	*HSP104* (4.45)	*MDJ1* (3.32)	*SIS1* (2.84)	*SSC1* (2.84)	*HSP78* (2.81)			
Mitotic spindle organization	*DAD1* (4.84)	*KIP1* (3.18)	*KIP3* (3.15)	*IPL1* (3.10)	*TUB4* (2.66)	*NUF2* (2.36)	*DAD2* (2.11)			
Protein refolding	*SSA2* (10.85)	*HSP70* (7.09)	*HSP60* (6.21)	*HSP104* (4.45)	*MDJ1* (3.32)	*SSC1* (2.84)	*HSP78* (2.81)			
Arginine biosynthetic process	*ARG8* (4.08)	*CPA1* (3.57)	*ARG4* (2.89)	ARG5%2C6 (2.73)	*ARG1* (2.63)	*ARG2* (2.28)				
Cellular response to nitrogen starvation	*MEP2* (20.17)	*MEP1* (4.75)	*AUT7* (3.07)	*APG7* (2.11)	*RHB1* (2.06)					
Histidine biosynthetic process	*HIS5* (7.56)	*HIS7* (3.01)	*HIS4* (2.34)	*HIS1* (2.23)						
Amine metabolic process	*AMO1* (3.60)	*AFP99* (2.32)	*AMO2* (2.10)							
Protein processing involved in protein targeting to mitochondrion	*MAS1* (2.73)	*AXL1* (2.69)	*MAS2* (2.66)							
Microtubule-based movement	*KIP1* (3.18)	*KIP3* (3.15)	*KAR3* (3.09)							
Cellular heat acclimation	*HSP104* (4.45)	*HSP21* (3.48)	*HSP78* (2.81)							
Positive regulation of mitochondrial translation	*MAM33* (2.07)									
